# Promotions

**Published:** 1781-05

**Authors:** 


### PROMOTIONS.

Dr. Henry Revell Reynolds, and Dr. Thomas Bowdler, phyſicians in London, to be Fellows of the Royal Society.—Dr. Martin Wall to be reader in chemiſtry at Oxford.—*April* 28. Mr. Valentine Jones to be ſurgeon to the firſt troop of horſe guards, in the room of Mr. Robert Sinclair.—*May* 26. Dr. Thomas Clerk, member of the College of Phyſicians, London, to be phyſician to the forces in North America, under the command of Sir Henry Clinton.—Mr. Patrick trick Connor, late aſſiſtant ſurgeon to the hoſpital at St. Vincent's, to be ſurgeon to the hoſpital in the Leeward Iſlands.

